# Cognitive Performance and Long-Term Social Functioning in Psychotic Disorder: A Three-Year Follow-Up Study

**DOI:** 10.1371/journal.pone.0151299

**Published:** 2016-04-15

**Authors:** Claudia J. P. Simons, Agna A. Bartels-Velthuis, Gerdina H. M. Pijnenborg, Genetic Risk and Outcome of Psychosis (GROUP) Investigators

**Affiliations:** 1 GGzE, Institute for Mental Health Care Eindhoven and De Kempen, Eindhoven, The Netherlands; 2 Maastricht University Medical Centre, Department of Psychiatry and Psychology, School for Mental Health and Neuroscience, European Graduate School of Neuroscience (EURON), South Limburg Mental Health Research and Teaching Network (SEARCH), Maastricht, The Netherlands; 3 University of Groningen, University Medical Center Groningen, University Center for Psychiatry, Groningen, The Netherlands; 4 University of Groningen, Department of Clinical Psychology and Experimental Psychopathology, Groningen, The Netherlands; 5 GGZ Drenthe, Department of Psychotic Disorders, Assen, The Netherlands; Katholieke Universiteit Leuven, BELGIUM

## Abstract

**Objective:**

Studies have linked cognitive functioning to everyday social functioning in psychotic disorders, but the nature of the relationships between cognition, social cognition, symptoms, and social functioning remains unestablished. Modelling the contributions of non-social and social cognitive ability in the prediction of social functioning may help in more clearly defining therapeutic targets to improve functioning.

**Method:**

In a sample of 745 patients with a non-affective psychotic disorder, the associations between cognition and social cognition at baseline on the one hand, and self-reported social functioning three years later on the other, were analysed. First, case-control comparisons were conducted; associations were subsequently further explored in patients, investigating the potential mediating role of symptoms. Analyses were repeated in a subsample of 233 patients with recent-onset psychosis.

**Results:**

Information processing speed and immediate verbal memory were stronger associated with social functioning in patients than in healthy controls. Most cognition variables significantly predicted social functioning at follow-up, whereas social cognition was not associated with social functioning. Symptoms were robustly associated with follow-up social functioning, with negative symptoms fully mediating most associations between cognition and follow-up social functioning. Illness duration did not moderate the strength of the association between cognitive functioning and follow-up social functioning. No associations were found between (social) cognition and follow-up social functioning in patients with recent-onset psychosis.

**Conclusions:**

Although cognitive functioning is associated with later social functioning in psychotic disorder, its role in explaining social functioning outcome above negative symptoms appears only modest. In recent-onset psychosis, cognition may have a negligible role in predicting later social functioning. Moreover, social cognition tasks may not predict self-reported social functioning.

## Introduction

Although there is some variability in the literature, most studies suggest psychotic disorder is characterized by a stable, diminished performance on the majority of cognitive domains [1,2], which presents itself before the onset of psychosis, and becomes more pronounced around the first psychotic episode, so that cognitive performance drops from approximately 0.5 SD below the healthy control mean in the prodromal stage to 1 to 2 SD below the healthy control mean in the first episode [3,4]. However, the exact course and pattern of alteration in cognitive functioning, and its relationship to functional outcome, remain unclear.

The cognitive deficits associated with psychotic disorders are linked to everyday social functioning and may limit the rate of functional improvement [5]. Cognitive abilities [6] and social cognitive skills [7,8] are consistently associated with various domains of social functioning. Research suggests that cognitive abilities may have bottom-up causal influences on the acquisition of social or living skills and on the deployment of these skills in the real world [9] which influence social cognition, which in turn may influence social functioning [10,11]. Accumulating evidence supports the suggestion that social cognition may be a mediating link between cognition and social functioning (e.g. [11–13], although social cognition may also explain unique variance in social functioning despite non-social cognitive underpinnings [7].

Despite the large body of evidence linking cognitive abilities to social functioning, literature shows mixed findings for the relevance of cognitive functioning as a prognostic factor for social functioning, that is, independent of clinical symptoms and negative symptoms in particular [14–17]. Whereas some studies suggest that cognitive functioning and negative symptoms may be independent predictors of functional outcome, other studies suggest that a substantial part of the explained variance in functional outcome is shared between cognitive functioning and clinical symptoms [14–16], with negative symptoms at least partially mediating the association between cognition [16], respectively social cognition [14], and functional outcome.

Additionally, the predictive value of cognitive functioning in the early phase of psychosis is unclear [18,19]. Although cognitive deficits are mostly seen as a stable trait in psychotic disorder, conflicting findings exist regarding the magnitude of cognitive underperformance during the first episode of schizophrenia compared with chronic schizophrenia, and some studies have found evidence for further cognitive decline after the onset of psychosis in at least some cognitive domains [20]. Modelling the prospective relations between cognition, social cognition and social functioning, and investigating the impact of clinical symptoms on these associations, may help to define therapeutic targets to improve functioning. Yet, most studies investigated associations between social cognition and functional outcome at a cross-sectional level, few studies investigated mediating effects of social cognition in the model of cognition–functional outcome longitudinally [11,13,21], and most studies focused on chronic schizophrenia, without comparing cognition-functional outcome associations with a healthy control sample. Therefore, it is unclear whether processes associated with social functioning differ from that of healthy controls.

Furthermore, processes may not be generalizable across different stages of the illness. Illness duration and treatment effects may influence associations between cognitive functioning and functional outcome and prognostic factors may have different predictive values in early psychosis [18,19]. Thus, studying these associations in patient samples differing in duration of illness may have implications for selecting appropriate therapeutic targets at various stages of the disorder. A recent systematic review [18] provides tentative evidence that cognitive functioning may be prognostic of functional outcome in early psychosis, nevertheless a meta-analysis suggests that illness chronicity does not moderate the association between cognition and social functioning [7]. The limited amount of studies conducted in recent-onset psychosis and the mixed results in more chronic samples warrant further research.

The aim of the present study was to investigate the contribution of cognition and social cognition in the prediction of social functioning, by first comparing the longitudinal association between baseline cognitive and social cognitive performance and three-year follow-up social functioning in patients with psychotic disorder versus healthy controls; and subsequently by analysing the associations in patients, integrating symptoms in a longitudinal model to account for the mediating role clinical symptoms may play in the cognition-social cognition-functional outcome relationships. To further clarify the associations, cross-sectional analyses were presented to explore concurrent associations with social functioning. We also investigated whether associations between cognitive functioning and social functioning varied in strength depending on illness duration.

## Materials and Methods

### Sample and measures

Data derive from the baseline (T_0_) and first follow-up (T_1_) measurements of the longitudinal ‘Genetic Risk and Outcome in Psychosis’ (GROUP) study (see [22] for further details). At baseline (T_0_), the full GROUP sample consisted of 1,119 patients with non-affective psychotic disorder and 589 control subjects. Inclusion criteria were: (i) age range 16 to 50 years, (ii) diagnosis of non-affective psychotic disorder, (iii) first mental health care contact for psychotic symptoms no longer than 10 years ago, and (iv) good command of Dutch language. Control subjects had no first or second degree relative with a psychotic disorder as established by the Family Interview for Genetic Studies [23] with the control subject as the informant. DSM-IV diagnoses, assessed with the Comprehensive Assessment of Symptoms and History (CASH) interview [24] or Schedules for Clinical Assessment for Neuropsychiatry (SCAN 2.1) [25], were: schizophrenia and related disorders (n = 945, 84%), other psychotic disorders (n = 149, 13%), and psychotic illness in the context of substance abuse or somatic illness (n = 9, 1%). At T_1_, three years after baseline, the GROUP sample consisted of 804 patients with non-affective psychotic disorder and 462 healthy controls.

Current severity of clinical symptoms was assessed in patients with the Positive and Negative Syndrome Scale (PANSS; [26]). Recent-onset psychosis was defined at baseline as duration of illness ≤ 2 years, consistent with definitions in the literature [27–30].

The interviews and neuropsychological testing were conducted by research assistants (psychologists, psychiatrists, nurses and PhD students) who had received extensive and repeated training (see [22] for the assessment training procedure).

### Ethical statement

The study protocol was approved centrally by the standing ethics committee (Medisch Ethische Toetsingscommissie, UMC Utrecht) and was conducted in accordance with the Helsinki Declaration of 1975, as revised in 2008. All participants gave written informed consent. Written informed consent was also obtained from the parents or guardians of those aged 16–17 years.

### Cognitive functioning

All subjects were assessed with a cognitive test battery containing the following tasks: the Wechsler Adult Intelligence Scale-III Short form (IQ) [31] consisting of the subtests Digit Symbol–Coding (processing speed), Arithmetic (working memory), Block Design (reasoning and problem solving), and Information (acquired knowledge); the Continuous Performance Test-HQ (CPT) (attention/vigilance) [32]; the Word Learning Task (verbal learning and memory) [33]; and the Response Shifting Task (set-shifting), which is a modified version of the Competing Programs Task [34,35]. For the CPT, outcome variable was a sensitivity index (number of correct detections of targets minus the number of false alarms for non-target Q stimuli). For the Word Learning Task (WLT), immediate recall (number of words recalled over the three 15-word trials) and retention rate (delayed free recall after 20 minutes divided by the maximum score of immediate recall trials 1–3) were used. For the Response Shifting Task (RST), a set-shifting cost score was used (decrement in accuracy during a reversal response rule condition compared with an imitation response rule condition). Test performance in the GROUP study has been described previously [36].

### Social cognition

Baseline assessment included two dimensions of social cognition: emotion perception and theory of mind (ToM). Emotion perception was measured using the Degraded Facial Affect Recognition task (DFAR; [37]). Outcome measure was the overall proportion correct.

ToM was assessed using the Hinting Task, which assesses the mentalizing capacity required to comprehend real intentions behind indirect speech [38]. Outcome measure was the sum of the ten item scores (range 0–20). The Hinting Task was administered at T_0_ only.

### Social functioning

Self-reported social functioning was assessed with the Social Functioning Scale (SFS; [39]) at three-year follow-up. The SFS is a self-rating scale of community functioning of individuals with schizophrenia [39]. The scale consists of seven subscales: social engagement/withdrawal, interpersonal behaviour, independence—competence, independence—performance, recreation, pro-social behaviour, and employment. Outcome measure was the total score on the SFS calculated as the mean of the seven scaled subscale scores, with higher scores reflecting better social functioning (range 59.7–134.9). Cronbach’s alpha for these seven subscale scores was adequate (α = 0.80). The SFS was administered at T_1_ only.

### Statistical analysis

Regression analyses were run in Stata version 13 [40] with T_0_ cognition and T_0_ social cognition variables as independent variables and T_1_ (three-year follow-up) social functioning as dependent variable, using age and sex as *a priori* covariates. Given that some families contributed more than one subject, hierarchical clustering of data was taken into account by including a family as a random effect in a linear mixed model, using the stata xtmixed command, fitted with restricted maximum likelihood estimation.

First, case-control analyses were conducted in which the interaction between status (patient vs. healthy control) and T_0_ cognition and T_0_ social cognition was tested in the longitudinal model with T_1_ social functioning as dependent variable, adjusting for sex and age.

Next, the longitudinal associations between cognition, social cognition, and social functioning were further explored in the patient sample, adjusting for sex and age. To further clarify T_0_-T_1_ longitudinal associations, T_1_-T_1_ cross-sectional analyses were presented to explore concurrent associations with social functioning.

To investigate whether cognition, social cognition, and symptoms may be independent predictors of social functioning or whether a mediational path can be distinguished, mediation analyses were planned using the Sobel–Goodman method (sgmediation in Stata). As advocated [41,42], we used non-parametric bootstrap resampling techniques to explore the indirect effects. In the case of significant main associations between (i) T_1_ social functioning–T_0_ cognition, and (ii) T_0_ social cognition–T_1_ social functioning, and/or T_0_ symptoms–T_1_ social functioning; each of the potential mediators (T_0_ social cognition and/or T_0_ symptoms) was added separately to the model of T_0_ cognition predicting T_1_ social functioning. The assumption-free bootstrap routine using case resampling (2000 iterations) was used to yield a percentile-based confidence interval for the indirect (i.e. mediated) effect.

Because associations between cognitive functioning and functional outcome may be influenced by illness phase and treatment effects, and prognostic factors may have different predictive values in early psychosis [18,19], illness duration was added to the model as a moderator, thus testing the cognitive measure × illness duration interaction. Furthermore, sensitivity analyses were conducted using the above mentioned linear regression models in recent-onset psychosis patients only.

Risk of type 1 error was addressed by applying Simes’ modification of the Bonferroni correction [43] to account for the multiple non-independent cognitive measures.

Release 3.02 of the GROUP database was used for the analyses.

## Results

### Sample

Of the 1,119 patients in the GROUP sample, 745 (67%) had three-year follow-up data for social functioning. Sample sizes for the main adjusted analyses ranged from n = 627–706. Of the 745 patients, 223 of were identified as patients with a recent onset psychosis. Of the 589 control subjects, 447 (76%) had follow-up data for social functioning. The control sample size for the comparison with the patient sample ranged from 398–462. Table 1 displays demographics of the patient and control sample and the clinical characteristics of the patient sample.

**Table 1:** Demographic and clinical characteristics.

	Patients				Controls			
	Mean	SD	range	n	Mean	SD	range	n
**Age (T**_**1**_**)**	30.2	7.2	18–59	742	34.1			443
**Sex, % male**	76.0%			745	44.1%			447
**Ethnicity, % white (Dutch/Belgian)**	84%			730	93%			439
**Residential status (%)**				677				413
single	42%				21%			
with parent(s)	24%				14%			
with partner/family	15%				61%			
sheltered living	14%				0%			
**Employment status, %**				656				
employed	74%							
none	16%							
study	7%							
household	3%							
**T**_**1**_ **Social functioning (SFS)**	112.5	9.3	84.6–134.9	745	123.9	5.6	99.5–133.6	447
**Baseline (T**_**0**_**) estimated IQ**	96.7	15.9	57–146	698	110.8	15.2	68–152	441
**PANSS symptoms**^b^								
positive	1.76	0.75	1–5.29	719				
negative	1.91	0.82	1–5.43	719				
general	1.69	0.51	1–3.69	719				
**Dose antipsychotic medication**^a^	7.5	33.4	0–676	744				
**Illness duration (in years)**	4.5			713				
**Recent-onset psychosis (illness ≤2 yrs)**	31.3%	4.1	0.2–41.1	713				

SFS = social functioning scale, PANSS = positive and negative syndrome scale

^a^ in milligrams haloperidol equivalents

^b^ Mean scores on three factors (positive, negative, and general symptoms) of the Positive And Negative Syndrome Scale (PANSS)

Patients with missing values on the T_0_ cognition, social cognition, or PANSS variables had similar T_1_ SFS scores compared with patients with non-missing values, with the exception that missing T_0_ CPT scores were significantly associated with lower T_1_ SFS scores. Patients with missing T_1_ social functioning scores had significantly lower scores on T_0_ cognition (except for RST and DFAR) and T_0_ social cognition variables and fewer T_0_ symptoms than patients with T_1_ social functioning data. S1 Table gives on overview of the demographic and clinical characteristic of included patients compared with patients with no T_1_ social functioning data.

### Case-control

Patients had significantly lower T_1_ SFS scores than healthy control subjects (B = -10.62, 95% CI -11.66;-9.57, *p*<0.001) and had significantly lower T_0_ cognitive and social cognitive scores (*p*’s<0.001).

Status (patient vs. control) significantly moderated the association between T_0_ performance and T_1_ social functioning for T_0_ Digit Symbol–Coding (B = 0.06, 95% CI 0.005;0.12, *p* = 0.03) and T_0_ verbal memory immediate recall (B = 0.17, 95% CI 0.004;0.33, *p* = 0.04), suggesting that the association between performance on these tasks and follow-up social functioning was stronger in patients than in control subjects. For the other T_0_ (social) cognitive measures the interaction with Status was non-significant (*p*’s>0.10).

### Predictors of social functioning in patients

Table 2 presents the results for the longitudinal analyses in patients. Except for verbal memory retention and the response shifting task, all cognitive measures were significantly associated with T_1_ social functioning, whereas the two social cognition variables were not significantly associated with T_1_ social functioning (Table 2). T_0_ PANSS positive, negative, and general symptoms were all significantly associated with T_1_ social functioning. For comparison, Table 3 gives the T_1_ cross-sectional associations.

**Table 2:** Longitudinal analyses: Regression analyses of baseline cognitive and social cognitive variables with three-year follow-up social functioning.

variable	B	95% CI	adjusted *p* ^a^
**Cognition**			
IQ	0.09	0.05; 0.14	<0.0001
Verbal memory immediate	0.19	0.08; 0.30	0.001
Verbal memory retention	-2.05	-5.32; 1.21	0.26
Attention/vigilance (CPT)	0.08	0.04; 0.12	0.001
Set-shifting (RST)	-1.16	-3.88; 1.57	0.43
Digit Symbol-Coding	0.11	0.07; 0.16	<0.0001
Arithmetic	0.30	0.16; 0.45	0.0001
Block Design	0.07	0.033; 0.11	0.0009
Information	0.21	0.08; 0.34	0.001
**Social cognition**			
Affect recognition	0.04	-0.02; 0.11	0.24
Hinting task	0.22	-0.04; 0.48	0.09
**PANSS symptoms**			
positive	-2.97	-3.86; -2.08	<0.0001
negative	-4.09	-4.87; -3.31	<0.0001
general	-5.49	-6.77; -4.21	<0.0001

CPT = continuous performance test, RST = response shifting task, DFAR = degraded facial affect recognition task, PANSS = positive and negative syndrome scale

^a^ *p*-value corrected for multiple testing using Simes’ modification of the Bonferroni correction

**Table 3:** Cross-sectional analyses: Regression analyses of three-year follow-up cognitive and social cognitive variables with three-year follow-up social functioning.

variable	B	95% CI	adjusted *p* ^a^
**Cognition**			
IQ	0.12	0.08; 0.16	<0.0001
Verbal memory immediate	0.27	0.16; 0.37	<0.0001
Verbal memory retention	6.39	2.96; 9.81	0.0003
Attention/vigilance (CPT)	0.06	0.01; 0.11	0.03
Set-shifting (RST)	-0.83	-3.66; 2.01	0.57
Digit Symbol-Coding	0.16	0.12; 0.20	<0.0001
Arithmetic	0.33	0.19; 0.47	<0.0001
Block Design	0.09	0.05; 0.13	<0.0001
Information	0.27	0.15; 0.38	<0.0001
**Social cognition**			
Affect recognition	0.06	-0.004; 0.13	0.07
Hinting task	N/A^b^		
**PANSS symptoms**			
positive	-4.15	-5.15; -3.15	<0.0001
negative	-6.21	-7.01; -5.40	<0.0001
general	-8.98	-10.35; -7.61	<0.0001

CPT = continuous performance test, RST = response shifting task, DFAR = degraded facial affect recognition task, PANSS = positive and negative syndrome scale

^a^ *p*-value corrected for multiple testing using Simes’ modification of the Bonferroni correction

^b^ Hinting task was not administered at T_1_, therefore no cross-sectional data are reported for this task

Mediator analyses were planned for the T_0_ cognitive tasks for which *p* <0.05 (all cognitive variables except verbal memory retention and RST) with T_0_ symptoms (positive, negative and general) as mediators. Mediation analyses were conducted to test for the possibility that T_0_ symptoms mediate the relationship between T_0_ cognitive functioning and follow-up social functioning. Each mediation model tested the effect of one mediator variable (T_0_ symptom dimension) on the relation between one independent variable (T_0_ cognitive variable) and T_1_ social functioning, with age and sex as covariates. Given that likelihood-ratio tests indicated that multilevel random effects models did not provide a better fit than simple linear regression models (*p*>0.10) for these data, Sobel-Goodmann tests were used to test for indirect effects in the simple linear regression models.

Table 4 shows the results for the bootstrapped mediation analyses. All T_0_ cognition—T_1_ social functioning associations were significantly mediated by symptoms, except positive symptoms did not mediate the association between T_0_ CPT performance and T_1_ social functioning. Positive and general symptoms only partially mediated the associations, whereas negative symptoms fully mediated the association between most T_0_ cognitive variables and T_1_ social functioning, with only Digit Symbol−Coding and CPT having a significant direct effect on T_1_ social functioning independent of negative symptoms. The proportion of explained variance from an adjusted R^2^ of 0.09 for the model including verbal memory immediate recall, positive symptoms, age and sex to an adjusted R^2^ of 0.16 for the model including Digit Symbol−Coding, negative symptoms, age and sex.

**Table 4:** Mediation analyses.

Cognitive predictor	Mediator	Direct effect		Indirect effect	
		B	95% CI	B	95% CI
**IQ**	Positive symptoms	0.07	0.03;0.11	0.02	0.01;0.03
	Negative symptoms	0.04	-0.002;0.08	0.05	0.03;0.07
	General symptoms	0.06	0.02;0.10	0.03	0.02;0.04
**Verbal memory immediate**	Positive symptoms	0.15	0.04;0.26	0.04	0.01;0.07
	Negative symptoms	0.09	-0.02; 0.19	0.10	0.06;0.15
	General symptoms	0.13	0.02;0.24	0.06	0.03;0.10
**Attention/Vigilance (CPT)**	Positive symptoms	0.07	0.03;0.11	0.01	-0.003;0.02
	Negative symptoms	0.04	0.001;0.08	0.04	0.02;0.06
	General symptoms	0.06	0.02;0.10	0.02	0.005;0.04
**Digit Symbol-Coding**	Positive symptoms	0.09	0.04;013	0.02	0.01;0.03
	Negative symptoms	0.06	0.01;0.10	0.05	0.03;0.07
	General symptoms	0.07	0.03;0.12	0.03	0.02;0.05
**Arithmetic**	Positive symptoms	0.23	0.08;0.37	0.07	0.03;011
	Negative symptoms	0.13	-0.01;0.28	0.17	0.11;0.22
	General symptoms	0.19	0.04;0.33	0.11	0.06;0.16
**Block Design**	Positive symptoms	0.05	0.01;0.09	0.02	0.01;0.09
	Negative symptoms	0.04	-0.003;0.08	0.03	0.02;0.05
	General symptoms	0.05	0.001;0.09	0.02	0.01;0.04
**Information**	Positive symptoms	0.16	0.04;0.29	0.04	0.01;0.07
	Negative symptoms	0.07	-0.05;019	0.13	0.08;0.19
	General symptoms	0.14	0.02;0.27	0.06	0.02;0.10

### Recent-onset psychosis

Illness duration (continuous variable) was not associated with T_1_ social functioning (B = -0.12, 95% CI -0.29;0.04, *p* = 0.15) and did not moderate the association between T_0_ cognitive measures and T_1_ social functioning (*p*’s>0.20).

Table 5 displays the results of the sensitivity analyses conducted in recent-onset patients only (illness duration at baseline ≤ 2 years). No significant longitudinal associations were found between cognition or social cognition on the one hand and three-year follow-up social functioning on the other. In contrast, baseline negative and general symptoms were a significant predictor of three-year follow-up social functioning.

**Table 5:** Sensitivity analyses of baseline cognitive and social cognitive variables with three-year follow-up social functioning in patients with recent onset psychosis.

variable	Longitudinal			Cross-sectional	
	b	95% CI	adjusted *p* ^a^	b	adjusted *p* ^a^
**Cognition**					
IQ	0.06	-0.02; 0.14	0.43	0.12	0.01
Verbal memory immediate	-0.03	-0.24; 0.19	0.86	0.08	0.46
Verbal memory retention	-1.86	-8.21; 4.49	0.86	7.05	0.06
Attention/vigilance (CPT)	0.05	-0.04; 0.14	0.48	0.08	0.19
set-shifting (RST)	-1.47	-7.55; 4.61	0.86	2.14	0.46
Digit Symbol-Coding	0.05	-0.03; 0.13	0.48	0.18	<0.0001
Arithmetic	0.29	0.02; 0.56	0.12	0.39	0.009
Block Design	0.04	-0.03; 0.12	0.48	0.06	0.18
Information	0.05	-0.20; 0.31	0.86	0.15	0.25
**Social cognition**					
Affect recognition (DFAR)	0.02	-0.11; 0.15	0.86	0.13	0.09
Hinting task	0.03	-0.46; 0.51	0.92	N/A^b^	N/A
**PANSS symptoms**					
Positive	-2.29	-4.09; -0.49	0.06	-4.64	0.0002
Negative	-4.64	-6.15; -3.12	<0.0001	-7.32	<0.0001
General	-4.60	-7.23; -1.96	0.005	-10.48	<0.0001

Analyses were *a priori* adjusted for age, sex, educational level (at T_0_ for longitudinal and T_1_ for cross-sectional analyses), antipsychotic dose ((at T_0_ for longitudinal and T_1_ for cross-sectional analyses)

CPT = continuous performance test, RST = response shifting task, DFAR = degraded facial affect recognition task, PANSS = positive and negative syndrome scale

^a^ *p*-value corrected for multiple testing using Simes’ modification of the Bonferroni correction

^b^ Hinting task was not administered at T_1_, therefore no cross-sectional data are reported for this task

## Discussion

We examined longitudinal associations between cognition, social cognition and social functioning in a large sample of patients with a psychotic disorder. A longitudinal association with three-year follow-up social functioning was found for most cognitive variables, with information processing speed and verbal memory immediate recall showing stronger associations with social functioning outcome in patients compared with healthy controls. Thus, the findings tentatively suggest that cognitive functioning may be a predictor for social functioning. Of all cognitive variables, information processing speed showed the largest association with social functioning (r = 0.22). The latter finding is in line with previous literature [44]. However, the proportion of the variance in social functioning that was explained by baseline cognitive functioning (<5%) or by the model of baseline cognitive functioning and negative symptoms (approximately 15%) is smaller than previous estimates of 25–50% as proportion of the variance that was explained by neurocognition alone[6], but larger than the percentage of 7.3% that was reported by Couture and colleagues for the model that included neurocognition as well as negative symptoms. At a cross-sectional level, cognitive functioning, associations between cognition and social functioning were larger than at the longitudinal level, but the proportion of the variance explained is still considerably smaller than the previous estimate of 25–50%. These findings are in line with Jabben and colleagues [17] who found weak cross-sectional associations between information processing speed in the absence of specific and unconfounded longitudinal associations. Also, most cognitive measures did not uniquely contribute to the explained variance, as results showed that symptoms and particularly negative symptoms −in line with previous literature [14,16,17,45]− meditated the association between cognitive functioning and social functioning. This may imply that factors such as a motivational deficits, social withdrawal, and impaired initiative may be underlying variables in the association between cognition and social functioning, which is in line with a previous study [46] suggesting that levels of intrinsic motivation are robustly and reliably associated with performance on cognitive tests, suggesting that shared motivation-cognition mechanisms should be investigated to enhance efforts to improve social functioning. In contrast to the other cognitive variables that were fully mediated by negative symptoms, information processing speed and attention/vigilance were only partly mediated and thus explained some of the variance in social functioning over and above negative symptoms.

Unexpectedly, none of the social cognitive measures was associated with self-reported social functioning at follow-up, in contrast with previous studies that reported a mediating role for social cognition (e.g. [11–13]), and with a recent study [47] that reported a mediating role for Hinting Task performance in the association between global cognition and self-reported daily functioning in patients with schizophrenia. Social cognition in the present sample was relatively mildly compromised, with affect recognition above impairment threshold (1 SD below control mean). Although the effect size for the Hinting Task was below 1 SD below control mean [36], patients were performing well above the level of patients in Couture and colleagues’ study [47]. This relatively good performance may explain why the association between Hinting Task performance and social functioning was stronger in Couture and colleagues’ study. Alternatively, the role of social cognition in predicting social functioning may not be robust, given that real-world social functioning is a complex construct that is likely determined by a complex interaction of several factors, including symptoms. Horan and colleagues [48] found social cognition to be predictive particularly for work functioning, whereas findings were less robust for the social functioning domain for which the predictive value was diminished after accounting for symptoms. In these first-episode patients, Horan and colleagues [48] found small and non-significant cross-sectional associations between social cognition and functional outcome at baseline, suggesting that clinical instability may have greater impact on social functioning than social cognitive skills. Social cognition research is hampered by inconsistent terminology and the lack of consensus on how to measure social cognition domains such as theory of mind and emotion perception, using well-validated, psychometrically sound instruments [49]. One important challenge is to construct a social cognition measure that is ecologically valid, that reflects the dynamic, interactive aspect of social skills applied to daily life social situations [50]. For example, the present measure of affect recognition, the DFAR, was not predictive of daily life social functioning in patients with psychotic disorders [51]. More ‘real-world’ indicators of social cognition may help enhance understanding of the path between cognitive performance and everyday social functioning, and may have the potential to improve current interventions to enhance functional improvement.

Illness duration did not significantly moderate the association between cognitive functioning and social functioning, suggesting that the associations are similar over the course of psychotic disorder. However, in contrast to the outcome in the patient group as a whole, sensitivity analyses in recent onset psychosis patients yielded inconclusive findings with respect to cognitive as longitudinal predictors of social functioning. In line with the latter findings, it has been suggested that cognition assessed within the first episode of the illness may not be a reliable predictor of functional outcome, explaining considerably less variance in outcome compared with previous cross-sectional studies with chronic patients [15]. Furthermore, a recent review showed that although most early psychosis studies did find an association between baseline cognitive functioning and follow-up functional outcome for at least one cognitive domain, more null associations than positive results were found for each cognitive domain [18]. A possible explanation for the lack of robust associations in recent onset samples may be that many psychosocial interventions are typically offered shortly after a first episode in early intervention programs and will therefore take place between baseline assessment and follow-up in this sub-sample. In this period, when illness course is very heterogeneous and several stages may be distinguished [52], patients may learn how to improve their daily functioning while compensating for cognitive impairments, or cognitive functioning may even improve with training. This may explain why associations between social functioning and cognition in the early stage of illness are few. Alternatively, the lack of associations in the recent-onset sample is in line with the findings in the complete sample that suggest that the amount of variance of social functioning explained by cognition is relatively small compared to previous findings.

The difference between the present results and previous findings may be related to differences in sample due to the recruitment procedures. Even though the majority of patients (84%) were diagnosed with schizophrenia-related disorders, the decision to include patients with other non-affective psychotic disorders may have attenuated effect sizes [36]. Furthermore, subjects were willing to participate in a demanding, longitudinal study protocol and may differ from subjects in research projects embedded within routine diagnostic or treatment procedures. Additionally, the study’s procedures to preferably include patients if at least one sibling was willing and able to participate, may have led to a selection excluding the more isolated and impaired patients. This may explain why overall cognitive deficits were relatively mild [36] compared with previous findings (e.g. [4,47]). It is possible that associations between cognitive functioning and social functioning may be more pronounced in samples that include only the more severe end of the spectrum of psychotic disorders or include patients in a more established phase of illness. Examining the longitudinal associations between relatively mild deficits and social functioning may explain why cognitive abilities failed to robustly predict social functioning, particularly in recent-onset patients where illness trajectories may be very heterogeneous [52].

### Limitations

The present study is unique in its naturalistic cohort design, with a broad neuropsychological assessment in a large sample of patients with a psychotic disorder and healthy controls. Nevertheless, some limitations need to be taken into account. First, baseline social functioning was not assessed; therefore, crossed-lagged analyses could not be performed. Because we did not have the possibility to evaluate this, we could not establish whether cognitive functioning may predict decline in or recovery of social functioning nor can we exclude that poor social functioning negatively impacts on cognitive performance rather than cognition impacting negatively on social functioning.

Second, follow-up data were not available for a substantial number of patients. Drop-outs had significantly poorer social and non-social cognitive skills than patients who remained in the study. Moreover, not all subjects had complete baseline cognitive test scores. Finally, a self-report questionnaire was used to assess functional outcome. Self-report is inevitably subjective and may be driven in part by within-person factors such as psychopathology. However, self-report may arguably still be better than observer-rated social behaviour because the latter assessment method also comes with a degree of interpretation and may result in a substantial extent of missing data.

The association between negative symptoms and social functional outcome might be partially explained by measurement overlap, e.g., the PANSS item Passive/Apathetic Social withdrawal has conceptual overlap with the SFS subscale social engagement/withdrawal. Measurement overlap may have resulted in an inflated correlation between negative symptoms and social functioning outcome [16] and may have resulted in an inflated indirect effect of negative symptoms on the association between cognitive functioning and social functioning.

Thus, although neuropsychological assessment gives an impression of current strengths and weaknesses, the results of the present study suggest that the prognostic value appears limited for most cognitive domains. Neuropsychological assessment may predict longer-term social functioning, but the present findings suggest that for most cognitive domains, the predictive value is modest and only information processing speed and attention/vigilance may provide additional prognostic information above assessing current symptom severity.

## Supporting Information

S1 Table Demographic and clinical characteristics of the included patients versus the patients with no follow-up social functioning scores. (DOCX)
