# Correction: Cognitive Performance and Long-Term Social Functioning in Psychotic Disorder: A Three-Year Follow-Up Study

**DOI:** 10.1371/journal.pone.0208347

**Published:** 2018-11-26

**Authors:** Claudia J. P. Simons, Agna A. Bartels-Velthuis, Gerdina H. M. Pijnenborg, Genetic Risk and Outcome of Psychosis (GROUP) Investigators

## Notice of republication

The original Supporting Information file Dataset 1 was published in error. The dataset was removed from the HTML and PDF versions of this article on November 15, 2018. The Data Availability statement has also been updated to reflect this change. Please download this article again to view the correct version.
