# Supplementary material for: Cognitive Performance and Long-Term Social Functioning in Psychotic Disorder: A Three-Year Follow-Up Study
Source: PLoS One. 2016 Apr 15;11(4):e0151299. doi: 10.1371/journal.pone.0151299 (PMC4833310; doi:10.1371/journal.pone.0151299)
Supplement: S1 Table — (DOCX) [file pone.0151299.s001.docx]

|  | Included patients | Patients with no follow-up data |  | |
| --- | --- | --- | --- | --- |
|  | Mean (SD) | Mean (SD) | Test statistic, *p*-value | |
| Age (T_0_)  Sex, % male  Baseline (T_0_) estimated IQ  PANSS^b^ positive  negative  general  Dose antipsychotic medication^a^  Illness duration (in years)  Recent-onset psychosis (illness ≤2 yrs) | 27.3 (7.3)  76.2%  96.7 (15.9)  1.76 (0.75)  1.91 (0.82)  1.69 (0.51)  7.5 (33.4)  4.5 (4.1)  31.3% | 28.2 (9.3)  75.9%  92.0 (15.7)  1.94 (0.78)  2.19 (0.91)  1.88 (0.55)  9.9 (90.5)  3.6 (3.4)  41.1% | t = -1.68  χ^2^ = 0.01  t = 4.42  t = -3.68  t = -4.87  t = -5.43  t = -0.64  t = 3.26  χ^2^ = 9.52 | 0.09  0.91  <0.0001  0.0002  <0.0001  <0.0001  0.53  0.001  0.002 |

Table S1. Demographic and clinical characteristics of the included patients versus the patients with no follow-up social functioning scores.
